# Establishment of a polymerase chain reaction-based method for strain-level management of *Enterococcus faecalis* EF-2001 using species-specific sequences identified by whole genome sequences

**DOI:** 10.3389/fmicb.2022.959063

**Published:** 2022-08-12

**Authors:** Hiroshi Hamamoto, Akihiko Ano Ogasawara, Masahiro Iwasa, Kazuhisa Sekimizu

**Affiliations:** ^1^Teikyo University Institute of Medical Mycology, Hachio-ji shi, Tokyo, Japan; ^2^Nihon Berm Co., Ltd., Chiyoda-ku, Tokyo, Japan; ^3^Drug Discoveries by Silkworm Models, Faculty of Pharma-Science, Teikyo University, Hachio-ji shi, Tokyo, Japan; ^4^Genome Pharmaceuticals Institute, Ltd., Bunkyo-ku, Tokyo, Japan

**Keywords:** strain management, probiotic, food production, whole-genome sequence, PCR

## Abstract

In the development and manufacture of fermented foods, it is crucial to control and manage the bacterial species used in the products. We previously reported a complete genome sequence analysis of the *Enterococcus faecalis* EF-2001 strain that was used for supplements. By comparing this sequence to the publicly available complete genome sequence of *E. faecalis* strains, we were able to identify specific sequences of the EF-2001 strain. We designed primer sets to amplify these specific regions and performed a polymerase chain reaction (PCR). We confirmed that the DNA fragments were specifically amplified in the genome of the EF-2001 strain, but not those of other lactic acid bacteria (LABs) or strains of the same genus. Furthermore, these primers amplified DNA fragments even in genomic DNA extracted from heat-treated bacteria at 121°C and foods containing the EF-2001 strain. These results suggest that this method allows for simple and highly accurate identification of specific fermentation strains, such as LABs at the strain level, which will be useful for controlling the quality of fermented foods.

## Introduction

In recent years, probiotics, such as lactic acid bacteria (LABs), were demonstrated to have favorable effects on the host health status, such as suppression of inflammatory responses (von Schillde et al., 2012) and reduction of immune-mediated neurologic symptoms (Sgritta et al., 2019). Some LABs also play an important role in food manufacturing by producing bacteriocins, such as nisin (Cleveland et al., 2001), and inhibiting the growth of pathogens, such as antimicrobial-resistant bacteria (Kaur et al., 2014). The phenotype of LAB differs among strains even in the same species (Nishida et al., 2016); thus, management and confirmation of the strains at the strain level are important in the manufacturing of food products. To date, the methods proposed for identifying LAB strains include a ribotyping method using pulsed-field gel electrophoresis (Tynkkynen et al., 1999), a triplicate arbitrarily primed-polymerase chain reaction (PCR) method (Cusick and O’Sullivan, 2000), and sequencing of hypervariable regions, such as clustered regularly interspaced short palindromic repeats (CRISPER) (Barrangou and Horvath, 2012). These methods, however, are complicated. Pulsed-field gel electrophoresis requires special equipment and has limited feasibility because it is based on band patterns. In addition, the method is difficult to apply to bacterial cells in a manufactured product. The establishment of a simpler and more accurate method is therefore desired. Regarding these points, PCR-based method is simple, easier, and low-cost, and it gives robust results (Treven, 2015). To establish this method, identification of strain-specific regions on a genome of a specific strain is required. Recently, the sequencing cost of the whole genome has been reduced, and strategies to identify strain-specific sequences have been proposed by comparative genomic analysis (Johansen et al., 2014; Perez-Lago et al., 2017).

The *Enterococcus faecalis* EF-2001 strain is a LAB isolated from healthy human feces (Takahashi et al., 2019). The strain, even the inactivated bacterium after heat treatment, has several bioactivities, such as immunostimulant effects (Ou et al., 2011), anti-inflammatory effects (Ishijima et al., 2014), and antidepressant effects (Takahashi et al., 2019), and has therefore been developed into a marketed food for human consumption. We analyzed the complete genome sequence of the EF-2001 strain by the hybrid assembly and found that the pathogenic island region is not present in this strain (Panthee et al., 2021). Based on the results of this sequence analysis, we hoped to establish a simple system for confirming the strain’s identity by determining the genomic regions that specifically exist in the genome of the EF-2001 strain and amplifying these regions by PCR. In this study, we mapped short reads obtained from whole genome analysis of the EF-2001 strain against the complete whole genome sequences of 42 strains publicly available in the genome database and collected unmapped reads to perform re-assembly and identify specific sequences of the EF-2001 strain. We then designed primer sets against these regions for PCR and confirmed that they were specific to the EF-2001 strain among LAB strains, including other *E. faecalis* strains. Our results indicate that strain-specific sequence identification based on whole genome analysis is useful for managing LAB strains in manufactured products.

## Materials and methods

### Identification of EF-2001 strain-specific sequence

Short reads (2,918,486 reads, average length 214 bp) obtained in a previous study using an Ion PGM sequencer (Thermo Fisher Scientific, MA, United States) (Panthee et al., 2021) were mapped on whole genome sequences of *E. faecalis* strains listed in Supplementary Table 1 using CLC Genomics Workbench ver. 12.0 (Qiagen, Aarhus C, Denmark) by suffix array algorism (Shrestha et al., 2014) with the following parameters: length fraction: 0.5 and similarity fraction: 0.8, mapped randomly for non-specific mapping reads. The unmapped reads were collected and assembled *de novo* by de Bruijn graphs (Gnerre et al., 2011) with the following parameters: minimum contig length: 250 bp, mismatch cost: 2, insertion cost: 3, deletion cost: 3, length fraction: 0.5, and similarity fraction: 0.8. We selected the contigs longer than 1 kb and confirmed that these contigs were unique to the EF-2001 strain or only a few strains harbored a similar sequence by the Basic Local Alignment Search Tool (BLAST) search of the National Center for Biotechnology Information (NCBI) database. Analysis of phage regions was performed as previously described using the PHAge Search Tool Enhanced Release (PHASTER) (Arndt et al., 2016; Panthee et al., 2021).

### Bacterial strains and culture conditions

#### Bacteria

We obtained the following strains from the NITE Biological Resource Center (NBRC) in Japan: *E. faecalis* NBRC3971, *E. faecalis* NBRC3989, *E. faecalis* NBRC12970, *E. faecalis* NBRC100480, *E. faecalis* NBRC100482, *E. faecalis* NBRC100483, *E. faecalis* NBRC100484, *Lactobacillus delbrueckii* subsp. *lactis* NBRC102622, *Lacticaseibacillus paracasei* subsp. *tolerans* NBRC15906, *Lactiplantibacillus plantarum* subsp. *plantarum* NBRC15891, *Lactobacillus acidophilus* NBRC13951, and *Lacticaseibacillus rhamnosus* NBRC3425.

#### Culture conditions

Lactic acid bacteria strains were grown on Lactobacilli De Man, Rogosa and Sharpe (MRS) Broth supplemented with 1.5% agar overnight at 37°C. Growing colonies were inoculated into MRS medium and cultured at 37°C overnight with shaking at 150 rpm.

#### Heat treatment of culture

Full growth cultures of bacteria were washed two times with saline by centrifugation at 12,000 × *g* for 5 min and suspended in 5 ml of saline. In total, 1 ml of suspensions was heated at the indicated temperature for 25 min with the water bath.

#### Chromosomal DNA extraction

DNAs were extracted from 20 μl of each strain culture incubated with the MRS medium at 37°C overnight using a Kaneka Easy DNA Extraction Kit, version 2 (Kaneka Co., Ltd., Hyogo, Japan) according to the instructions of the manufacturer. DNA extraction from heat-treated culture was performed with 200 μl, samples and DNA extraction from food was performed with 100 μl samples suspended in saline to 10 mg/ml.

#### Polymerase chain reaction conditions and agarose gel electrophoresis

Polymerase chain reaction was performed using KOD FX Neo (TOYOBO Co., Ltd., Osaka, Japan) following the instructions of the manufacturer under the following conditions: 10 s at 94°C, followed by 25 cycles at 98°C for 10 s, 60°C for 10 s, and 68°C for 30 s. Primers were mixed with the final 0.3 μM of each of the forward primers and two reverse primers (short and long; Table 1), and 10 ng of DNA template was used. The PCR products were analyzed by agarose gel electrophoresis using 1.4% agarose gel supplemented with 0.01% GelRed Nucleic Acid Gel Stain (Fujifilm Co., Ltd., Tokyo, Japan).

**TABLE 1:** Primer list for amplification of the EF-2001 genome.

Contig name		Forward primer		Reverse primer		Product size [base pair]
		Name	Sequence	Name	Sequence	
Contig_01	Long	EFC01_For	CGAAAAGGATGTAGTCAGCGG	EFC01_Rev. L	CCGAAGGCGAAACAGAGGAT	581
	Short			EFC01_Rev. S	CCCAGACATAATCGCATGGC	210
Contig_04	Long	EFC04_For	GCGGCTGCACAATTTATTGC	EFC04_Rev. L	AGAATACTTGGGCGGTCGTG	594
	Short			EFC04_Rev. S	AATTCAGCTTCGCTAGATAAGGC	150
Contig_07	Long	EFC07_For	CGCGTATGACTTGCAATCGA	EFC07_Rev. L	AGGATTGTTTGACGGTGCAA	510
	Short			EFC07_Rev. S	ACATGAGATAGTTGGGGTAGACA	183
Contig_11	Long	EFC11_For	CTTCAGAGAGCTGGGCGAAG	EFC11_Rev. L	TACTTTTTAGCTGCCCGCCC	513
	Short			EFC11_Rev. S	GGGTTGTAGCCCTACCCGAT	246
Contig_13	Long	EFC13_For	CGTAACGTGACATTGCGGAC	EFC13_Rev. L	ATGCCAGTACGTCGCGTTAA	600
	Short			EFC13_Rev. S	CGATTGTCAACTAATTGTGCCGA	206
Contig_16	Long	EFC16_For	CATGGCTTGCCGTTTCACAA	EFC16_Rev. L	ACCGCAACAACTACATACTACCA	509
	Short			EFC16_Rev. S	ACCAAAAGGAACGCTACCAGT	179
Contig_22	Long	EFC22_For	TCAGCATAATCCCCAGACGT	EFC22_Rev. L	AATGAACGCCCTTCAGCAGA	532
	Short			EFC22_Rev. S	GGCTCCTCTACCTGAACAAACT	147
Contig_25	Long	EFC25_For	GCGTTCAAACTGTTCTGGTGT	EFC25_Rev. L	TACAAGGCTTGCGAGGTAGC	548
	Short			EFC25_Rev. S	GCTGCAATGGAAAGCAAATCG	171
Contig_31	Long	EFC31_For	AGGCATATGGGTCATCTGCT	EFC31_Rev. L	GAGCATCACAGAGCCTCGAA	575
	Short			EFC31_Rev. S	AGAGATTTTTCAGTATTGCTGGGT	118
Contig_43	Long	EFC43_For	CGTTGGGTGTGCAGAAATGG	EFC43_Rev. L	TGTACCGTCAACCTCGTTCG	593
	Short			EFC43_Rev. S	AACGGGTTGCGACTCTTTTT	194
Positive control		EFPC01_For	AACGCTTCTTTCCTCCCGAG	EFPC01_Rev	GTGTCTCAGTCCCAGTGTGG	284
Universal primer set		9F	GAGTTTGATCCTGGCTCAG	1541R	AAGGAGGTGATCCAGCC	1554

## Results

### Identification of EF-2001 strain-specific sequences

**FIGURE 1:**
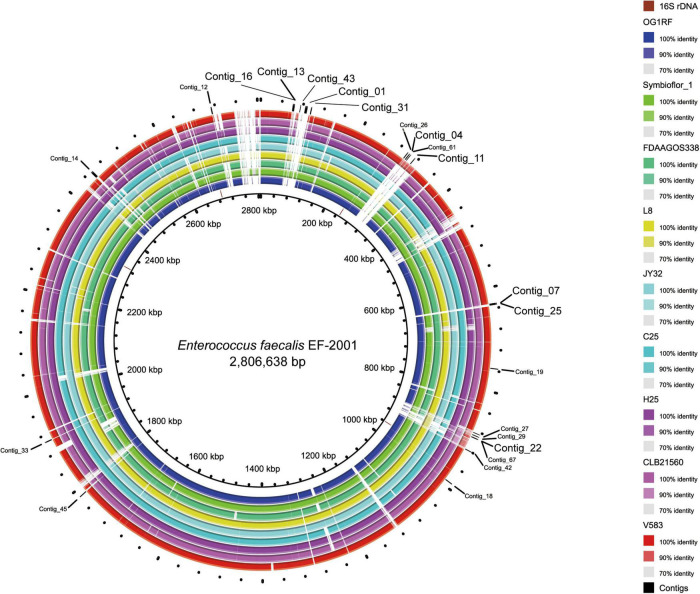
Location of specific sequences of the EF-2001 strain identified in this study. The figure is drawn by the Basic Local Alignment Search Tool (BLAST) Ring Image Generator (Alikhan et al., 2011) using the sequences of 9 *E. faecalis* strains. The innermost ring (black circle) shows the complete genome sequence of the EF-2001 strain, and the second ring from the inside shows the 16S rDNA sequence of the EF-2001 strain. The 3rd to 11th rings show genome sequences of *E. faecalis* OG1RF, Symbioflor 1, FDAAGOS338, L8, JY32, C25, H25, CLB21560, and V583 strain, respectively, and similarities with the EF-2001 strain are shown with each indicated color. The outermost ring shows a sequence specific to the EF-2001 strain.

We previously reported that the full-length genome of the EF-2001 strain was obtained by the hybrid assembly (Panthee et al., 2021). In that study, we used the short reads obtained to collect unmapped reads against the whole genome sequences of 42 strains, which were reported at the time of the initial analysis (Supplementary Table 1). Consequently, we obtained 69,137 unmapped reads (total 1.3 Mb) from 2.9 million reads (total 63 Mb). Using these unmapped reads, we performed *de novo* assembly and selected sequences longer than 1 kb, yielding 27 contigs (Figure 1). Among these contigs, we selected 10 contigs containing no plasmid sequences and with little homology to the public genomic sequence of other *E. faecalis* strains at the time of the primer design. Contigs 01, 13, 16, 31, and 43 belong to the region of PHAGE_Entero_phiFL2A_NC_013643(14); contigs 04 and 11 belong to region of PHAGE_Entero_ phiFL4A_NC_013644(43), and contig 22 belongs to region of PHAGE_Entero_phiFL1A_NC_013646(10). At present, no strain harboring all these phages has been deposited in the NCBI genome database. Contigs 07 and 25 are regions with unknown genes that exist specifically in the EF-2001 strain outside the phage region.

### Primer design and amplification of the EF-2001-specific region

**FIGURE 2:**
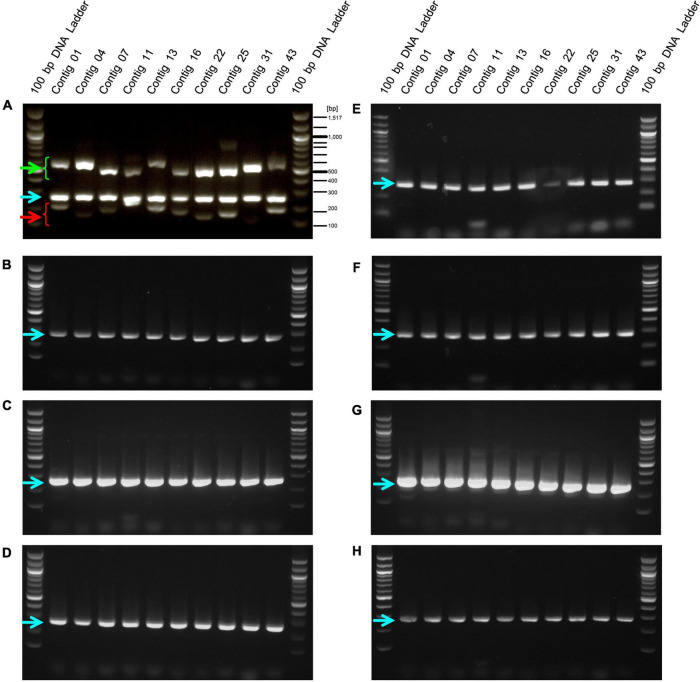
Amplification of DNA fragments by PCR with EF-2001 strain-specific primers against genomic DNA from different *E. faecalis* strains. PCR was performed using genome DNA extracted from **(A)** *E. faecalis* EF-2001, **(B)** *E. faecalis* NBRC3971, **(C)** *E. faecalis* NBRC3989, **(D)** *E. faecalis* NBRC12970, **(E)** *E. faecalis* NBRC100480, **(F)** *E. faecalis* NBRC100482, **(G)** *E. faecalis* NBRC100483, and **(H)** *E. faecalis* NBRC100484 with the short, long, and positive control primer sets listed in Table 1. Green and red arrowheads indicate bands that were amplified by the primer sets specific for the EF-2001 strain. The blue arrowhead indicates bands that were specifically amplified from the 16S rDNA sequence of *E. faecalis*.

**FIGURE 3:**
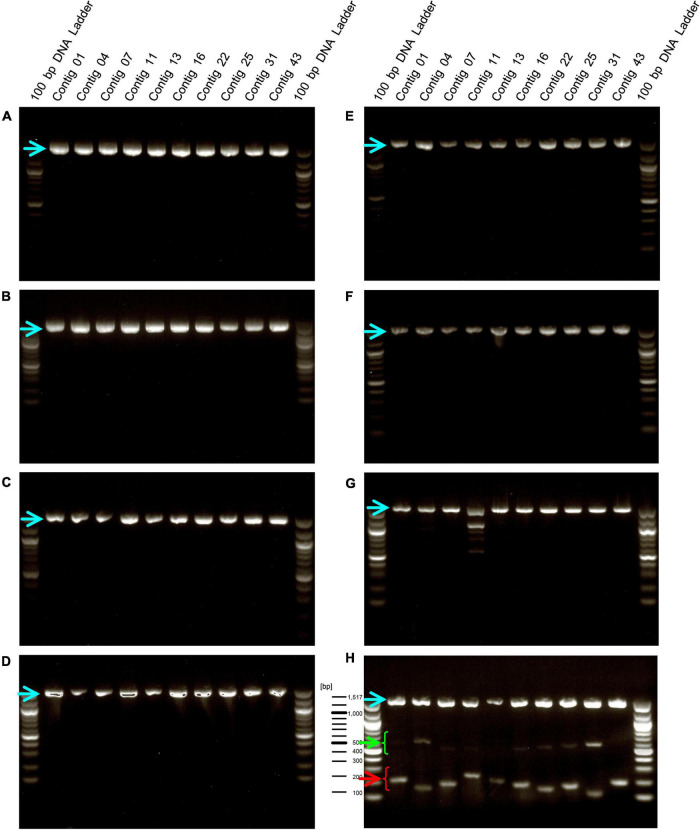
Amplification of DNA fragments by PCR using genomic DNA from other lactic acid bacteria (LAB) species. PCR was performed using genome DNA extracted from *E. faecium* Aus0004 **(A)**, *E. gallinarum* LMG13129 **(B)**, *L. rhamnosus* NBRC 3425 **(C)**, *L. acidophilus* NBRC 13951 **(D)**, *L. plantarum* subsp. *plantarum* NBRC 15891 **(E)**, *L. paracasei* subsp. *tolerans* NBRC 15906 **(F)**, *L. delbrueckii* subsp. *lactis* NBRC102622 **(G)**, and *E. faecalis* EF-2001 **(H)** with each short- and long primers with universal primer set for 16S rDNA, instead of a positive control primer set. Green and red arrowheads indicate bands that were amplified by the primer sets specific for the EF-2001 strain. The blue arrowhead indicates bands that were specifically amplified from the 16S rDNA of each strain.

We designed primers to amplify the above 10 selected contig regions (Table 1). The primers to amplify short DNA fragments were designed at the same time because the bacterial genome would be fragmented by longer heating times when lactobacilli are processed into foods and longer DNA fragments cannot be amplified (Suyama and Kawaharasaki, 2013). Further, we designed primers that amplify the 16s rDNA sequence of *E. faecalis* as a positive control to confirm that the strain was indeed *E. faecalis*. In this analysis, genomic DNA was extracted from 8 *E. faecalis* strains, including the EF-2001 strain, and DNA fragments were amplified by PCR using these primers. We found that all amplified DNA fragments of the EF-2001 strain were detected at the predicted size (Figure 2). On the other hand, DNA fragments derived from *E. faecalis*-specific rDNA sequences were detected in different *E. faecalis* strains, although no DNA fragments were found in the positions expected from the sequences present in the EF-2001 strain (Figure 2). Further, we tested LAB strains other than *E. faecalis*, such as *E. faecium, E. gallinarum, L. rhamnosus, L. acidophilus, L. plantarum* subsp. *plantarum, L. paracasei* subsp. *tolerans*, and *L. delbrueckii* subsp. *lactis*. We performed PCR using the primer sets shown in Table 1 using the universal primer set as a control and found that no DNA fragments were detected at any of the expected positions (Figure 3), although the bands originating from universal primer were detected in all strain except for the contig 11 of *L. delbrueckii* subsp. *lactis* NBRC102622. The results suggest that these primer sets are specific to genomic DNA of the EF-2001 strain, and they can thus be used to identify whether or not the bacteria in a sample, such as food product, is *E. faecalis* EF-2001 strain.

### Detection of the EF-2001 strain from food products

**FIGURE 4:**
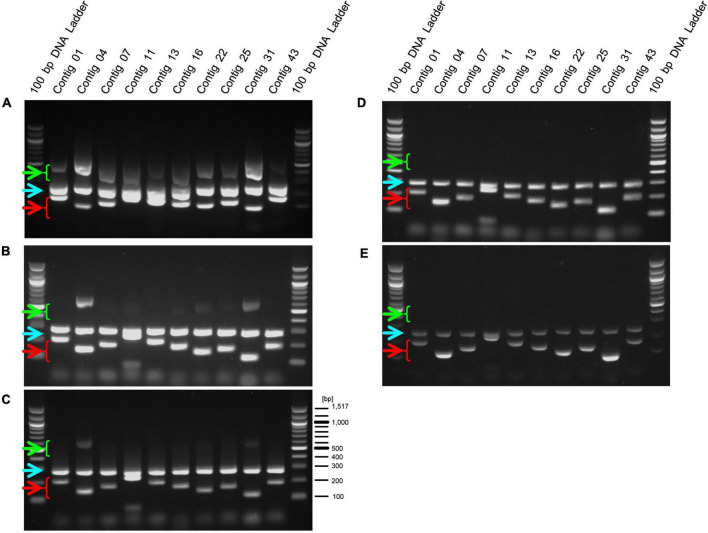
Amplification of DNA fragments by PCR using genomic DNA extracted from heat-treated cells. After heat-treatment of the EF-2001 cultured cells, genomic DNA was extracted, and PCR was performed with the short, long, and positive control primer sets. Panels **(A–E)** shows DNA that was untreated or treated at 70, 90, 110, and 120°C, respectively. Green arrowheads indicate DNA fragments amplified by primer sets (for longer fragments) and red arrowheads indicate DNA fragments amplified with primer sets (for shorter fragments). Blue arrowheads indicate DNA fragments that were specifically amplified from the 16S rDNA sequence of *E. faecalis*.

**FIGURE 5:**
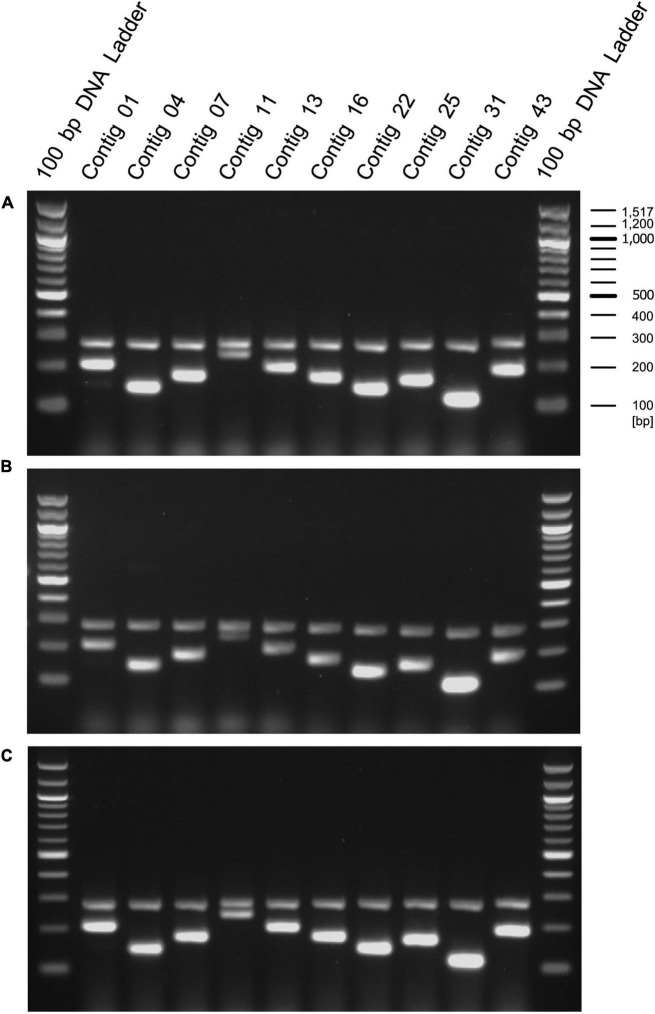
Amplification of DNA fragments by primed-polymerase chain reaction (PCR) using genomic DNA extracted from food products including EF-2001 strain. Genomic DNAs were extracted from health food products containing **(A)** 5 × 10^11^ cells per pack (BeRM Bien V75, Bien Co., Ltd.), sugar, and starch, **(B)** 1 × 10^12^ cells per pack, sugar, starch, and organic acid (BRM-A 150, Kaneroku Pharma Co., Lp.), and **(C)** 1.5 × 10^12^ cells per pack, sugar, starch, organic acid, mineral, and fragrance (TriEF, TRI-ENCE, Co., Ltd.), and PCR was performed using the short, long, and positive control primer sets.

Lactic acid bacteria are often used in food products that have been heat treated to prevent bacterial growth and to preserve the products over a long period of time (Adams, 2010; Pique et al., 2019). Therefore, we performed PCR using this primer set on genomic DNA extracted from the heat-treated EF-2001 strain. We found that amplification of the longer size DNA fragments was reduced in a temperature-dependent manner, and the fragments were not observed at temperatures above 110°C. In contrast, short DNA fragments were detected even after treatment at 120°C (Figure 4, green and red arrowheads). Furthermore, we conducted PCR on genomic DNAs extracted from three commercial products in which the heat-treated EF-2001 strain was used. We used the long- and short primer set with the positive control for 16S rDNA of *E. faecalis*, although, the expected lengths of 16S rDNA gene-derived fragments and shorter size DNA fragments were detected in all the products (Figure 5). From these results, these products contained the correct EF-2001 strain, and the bacteria were adequately heat treated.

## Discussion

Strain management is a critical issue for controlling the quality of fermented foods and supplements. Bacterial strain identification methods have been established for the management of LAB strains, such as the comparison of 16S rDNA sequences (Woese and Fox, 1977; Woese et al., 1990), genetic polymorphism of housekeeping genes (Ruiz-Garbajosa et al., 2006), DNA/DNA hybrid methods (De Ley et al., 1970), and complete gene sequence analysis (Panthee et al., 2021). Despite the availability of these methods, the quality of fermented food products is usually judged by differences in the physiologic and biochemical properties of the colony, such as the morphologic characteristics, sugar chaptalization, and fermentation product yield of each sugar. Identification of species by 16s rDNA sequences is performed because of its convenience, although it is not possible to confirm strain level. In this case, it is difficult to detect contamination by the same species. In contrast to these other methods, our method requires whole genome sequencing, but only once. Subsequently, the bacteria can be identified at the strain level using a simple and inexpensive PCR method with multiple primer sets targeting sequences specific to the strain. This method allows for highly accurate strain management. Until now, RT-PCR-based method targeting single nucleotide polymorphism or small deletion in the specific genes, CRISPER spacers (Shehata et al., 2021), were established. Compared to these methods, our method is easy to identify the EF-2001 strain-specific region.

In the present study, we identified EF-2001 strain-specific sequences using short reads obtained by previous whole genome analysis (Panthee et al., 2021) by collecting reads that were not mapped on the public complete genome sequence of other *E. faecalis* strains. Of the 10 contigs specific to the EF-2001 strain identified in this study, eight were originated from the phage genome region. Phages infecting bacteria also affect the properties of the bacteria by introducing foreign genes, such as drug resistance genes (Diaz-Munoz and Koskella, 2014; Torres-Barcelo, 2018). Therefore, these regions derived from the phage genome would be involved in the functionality of the EF-2001 strain itself, and it is important to confirm by PCR that no phage region dropouts have occurred. Many kinds of bacteriophage genomes have been identified in *E. faecalis* (Duerkop et al., 2014). Even if they are classified into the same class of phage, their sequences are not the same due to genome mutation or organization. In addition, bacterial strains rarely share the same combinations of bacteriophage types when they have multiple phages on their genome. Some of the regions amplified in this study are found in strains with very similar sequences that have recently been registered into the genomic database after the primers were designed (Supplementary Table 2), nevertheless, we found no strains possessing all sequences used to identify the EF-2001 strain. In addition, there is no strain registered that harbors the sequence of contig 04 and contig 11, so far. Therefore, the probability of incorrectly identifying strains other than EF-2001 strains using the primer sets listed in Table 1 is very low. Furthermore, our established method could detect the EF-2001 strain even from food products. Thus, this method can be used to identify bacteria at the strain level and can be applied to confirm strains used to produce foods or supplements. In addition, we prepared the primer sets for different size DNA amplification, allowing us to confirm the heat treatment of the bacteria using this system. In addition, it is also possible to quickly check contamination in the manufacturing process. In this system, contamination of other bacteria could be detected if the universal primer set for 16S rDNA, instead of the primer set for *E. faecalis-*specific 16S rDNA, as shown in Figure 3. In addition, this method is expected to be applicable for quality assurance to guarantee safety, as some subspecies of *E. faecalis* have virulence factors (Matsumoto et al., 2019). Further, this method is applicable to other LABs, because this method is not restricted to single nucleotide variation (SNP) or deletion of specific genes and is just required to perform whole genome sequence analysis.

## Data availability statement

The original contributions presented in this study are included in the article/Supplementary material, further inquiries can be directed to the corresponding author.

## Author contributions

HH conceived the idea, performed bioinformatic analysis, and wrote the manuscript. AO performed PCR analysis. MI made critical comments for experiments. KS critically revised the article for important intellectual content and provided final approval of the article. All authors contributed to the article and approved the submitted version.
