# Supplementary material for: Establishment of a polymerase chain reaction-based method for strain-level management of Enterococcus faecalis EF-2001 using species-specific sequences identified by whole genome sequences
Source: Front Microbiol. 2022 Aug 12;13:959063. doi: 10.3389/fmicb.2022.959063 (PMC9411961; doi:10.3389/fmicb.2022.959063)
Supplement: Supplementary file 1 [file Table_1.docx]

| Strain | Accession number | Genome size (bp) |
| --- | --- | --- |
| 62 | GCA_000211255.2 | 3,100,313 |
| ARO1/DG | GCA_002814115.1 | 2,878,753 |
| ATCC 29212 | GCA_000742975.1 | 3,048,131 |
| B594 | GCA_000391485.2 | 3,267,721 |
| BFFF11 | GCA_009685155.1 | 3,067,042 |
| C25 | GCA_005979285.1 | 2,958,621 |
| C54 | GCA_005979305.1 | 2,878,089 |
| CLB21560 | GCA_001989555.1 | 3,243,539 |
| CVM N48037F | GCA_004332015.1 | 3,151,142 |
| CVM N60443F | GCA_004332035.1 | 2,853,753 |
| D32 | GCA_000281195.1 | 3,062,505 |
| DENG1 | GCA_000550745.1 | 2,961,043 |
| DM01 | GCA_006766145.1 | 2,785,968 |
| FC | GCA_005158025.1 | 3,183,730 |
| FDAARGOS_324 | GCA_003030425.1 | 3,159,864 |
| FDAARGOS_338 | GCA_002208945.2 | 2,861,022 |
| FDAARGOS_528 | GCA_003812665.1 | 2,963,654 |
| FDAARGOS_611 | GCA_006364815.1 | 2,845,446 |
| FDAARGOS_324 | GCA_003030425.1 | 3,159,864 |
| HA-1 | GCA_006349345.1 | 2,955,738 |
| JY32 | GCA_010103655.1 | 2,933,718 |
| KB1 | GCA_001689055.2 | 3,026,009 |
| KUB3006 | GCA_003966385.1 | 3,138,474 |
| KUB3007 | GCA_003966405.1 | 3,171,737 |
| L12 | GCA_001886675.3 | 2,740,465 |
| L15 | GCA_009498155.1 | 2,998,025 |
| L8 | GCA_009498175.1 | 2,895,628 |
| LD33 | GCA_001598635.1 | 2,803,429 |
| MGYG-HGUT-01694 | GCA_902379005.2 | 2,810,675 |
| NCTC8732 | GCA_901553725.1 | 2,864,420 |
| NCTC8745 | GCA_900636775.1 | 2,719,199 |
| OG1RF | GCA_000172575.2 | 2,739,625 |
| R712 | GCA_009662495.1 | 3,117,418 |
| SGAir0397 | GCA_005484525.1 | 2,696,714 |
| sorialis | GCA_002163735.1 | 3,054,066 |
| Symbioflor 1 | GCA_000317915.1 | 2,810,675 |
| TY1 | GCA_003345275.1 | 3,200,228 |
| V583 | GCA_000007785.1 | 3,359,974 |
| VE14089 | GCA_006494835.1 | 3,238,854 |
| VE18379 | GCA_006494855.1 | 3,218,315 |
| VE18395 | GCA_006494875.1 | 3,201,501 |
| W11 | GCA_002355755.1 | 2,704,865 |

**Supplementary Table 1 Whole genome sequence used for mapping sequence reads originating from the EF-2001 strain**

| Contig No. | Stain name | | | | | | | | | | | | |
| --- | --- | --- | --- | --- | --- | --- | --- | --- | --- | --- | --- | --- | --- |
| Contig_01 | 732 | R5 | 26975 | 28099 | 28157 | 27688 | QZ076 |  |  |  |  |  |  |
| Contig_07 |  |  | 26975 | 28099 | 28157 |  |  |  |  |  |  |  |  |
| Contig_13 | 732 | R5 | 26975 | 28099 | 28157 | 27688 | QZ076 |  |  |  |  |  |  |
| Contig_16 | 732 | R5 | 26975 | 28099 | 28157 | 27688 | QZ076 |  |  | 142-1 | UAMS EL53 | UAMS EL54 | UAMS EL56 |
| Contig_22 |  |  |  |  | 28157 |  |  |  |  |  |  |  |  |
| Contig_25 |  |  | 26975 | 28099 | 28157 |  |  |  |  |  |  |  |  |
| Contig_31 |  |  | 26975 |  |  | 27688 |  | 111540047-1 | 111540027-2 |  |  |  |  |
| Contig_43 |  |  | 26975 |  |  | 27688 |  | 115400047-1 | 111540027-2 |  |  |  |  |

**Supplementary Table 2 Name of the strains that have a sequence very similar to the sequence to be amplified by PCR in this study**
